# Leveraging mHealth and Virtual Reality to Improve Cognition for Alzheimer’s Patients: A Systematic Review

**DOI:** 10.3390/healthcare10101845

**Published:** 2022-09-23

**Authors:** Clemens Scott Kruse, Keya Sen, Valery Armenta, Nicole Hubbard, Rebekah Brooks

**Affiliations:** School of Health Administration, Texas State University, San Marcos, TX 78666, USA

**Keywords:** mHealth, Alzheimer’s Disease, memory care

## Abstract

Background: Alzheimer’s Disease (AD) is a global problem affecting 58 million people, expected to reach a prevalence of 88 million people by 2050. The disease affects the brain, memory, cognition, language, and motor movement. Many interventions have sought to improve memory and cognition. mHealth and virtual reality (VR) are two such interventions. Objectives: To analyze studies from the last 10 years with older adults with AD to ascertain the effectiveness of telehealth techniques such as mHealth and VR for memory care. Methods: In accordance with the Kruse Protocol and reported in accordance with PRISMA 2020, five reviewers searched four research databases (PubMed, CINAHL, Web of Science, and ScienceDirect) on 3 August 2022 for studies with strong methodologies that fit the objective statement. Results: Twenty-two studies from 13 countries were analyzed for trends. Four interventions (mHealth/eHealth, VR, mHealth + VR, game console, and telephone) used RCT, quasi-experimental, pre-post, observational, and mixed methods. These interventions improved cognition, memory, brain activity, language, depression, attention, vitality, quality of life, cortical atrophy, cerebral blood flow, neuro plasticity, and mental health. Only three interventions reported either no improvements or no statistically significant improvements. Cost, time, training, and low reimbursement were barriers to the adoption of these interventions. Conclusion: mHealth and VR offer interventions with positive effectiveness for memory care for AD. The long-term effect of this improvement is unclear. Additional research is needed in this area to establish clinical practice guidelines.

## 1. Introduction

### 1.1. Rationale

Alzheimer’s Disease (AD) is a growing condition around the word. As we approached the COVID-19 pandemic, AD was the largest killer of older adults: it kills more people than breast cancer and prostate cancer [1]. The prevalence of the disease was calculated in 2021 to be 58 million people, but it is predicted to exceed 88 million by 2050 [1]. Of the dementia population, AD accounts for about 2/3 s [1]. There is currently no cure for AD, and there are only about 10 pharmaceuticals approved to manage the condition. The disease creates plaque on the brain (tau) that eventually affects the communication of 100 billion neurons in the brain, degrading and ultimately destroying these neurons [2]. Early stages of AD is seen as simple forgetfulness of recently learned facts, but late stages of AD affects speech, motor skills, and long-term memory [1]. Researchers and practitioners do not fully understand the etiology and pathogenesis of AD: we can treat the symptoms, but we cannot prevent or cure the disease [3,4,5]. Researchers have searched for decades for interventions to improve symptoms of cognitive decline, and one of these is cognitive training through telemedicine.

Many tests are used to assess impairment and symptoms associated with AD. AD affects cognition, which is a complex process in the brain that involves memory, abstraction and iconic concepts, mental operations, consciousness, search strategies, problem solving, and social context [6]. One common method to measure cognition is the mini-mental state examination (MMSE), which estimates a severity of cognitive impairment through a series of questions organized into seven categories: orientation to time, orientation to place, registration of three words, attention to calculation, recall of three words, language, and visual construction [7]. Given over time, the MMSE can identify rate of decline or document improvement.

Telemedicine is defined as healing from a distance using information communication technology to overcome geographical boundaries and increase health outcomes [8]. mHealth is a subset of telemedicine that leverages mobile technology to deliver some sort of intervention or interaction with a provider. mHealth interventions with patients who have AD suffer from barriers such as cognition, perception, physical ability, frame of mind, speech and language [9]. mHealth design must break steps into very simple, easy to understand modules, must often repeat instructions to keep the attention of the users, and use simple memory tests to avoid overwhelming the user [10]. mHealth has been coupled with other interventions such as transcranial alternating current during cognitive training, but results are not conclusive [11]. Virtual reality (VR) has also entered the area of AD research, specifically in the area of cognitive training. The reason is that VR exercises multiple perception components of psychophysics (visual, tactile, and kinesthetic perceptual sensations) [12]. The proponents of VR like its immersive and adaptable environment. It has been used in the areas of brain damage, poststroke intervention, musculoskeletal recovery, and in cognitive training for AD. This review will focus on the telemedicine-related interventions (mHealth, VR, and serious games) in the area of memory for AD patients. Multiple systematic literature reviews have examined this interaction. Many conclude that telemedicine can assess cognition, monitor activity, and improve communication with provider teams [13]. Telemedicine can positively affect mood, function, and quality of life, but its effect on cognition is unclear [14].

A systematic literature review and meta-analysis was published in 2022 that analyzed 16 Randomized Controlled Trials (RCTs) [15]. The meta-analysis focused on a smaller set of studies. It found that serious games are as effective as no intervention or passive interventions at improving executive functions. It concluded that conventional exercises were just as effective. The reviewers felt their group for analysis was too small for final conclusions.

A systematic literature review was published in 2022 that analyzed 28 studies over 10 years [9]. It evaluated several aspects of mHealth. It found positive perceptions of the users of mHealth (both AD patients and their caregivers). The caregivers attributed positive effect of mHealth interventions on their physical and mental health; however, effectiveness was not evaluated.

### 1.2. Objectives

The purpose of this review is to analyze the effectiveness of telemedicine-related interventions (mHealth, VR, and serious games) to improve cognition for older adults suffering from Alzheimer’s Disease or mild cognitive impairment (MCI) using published literature from the last 10 years. Secondary outcomes will be memory, language, mood, vitality, attention, brain waves, and other conditions measured and reported in the literature. Our review will be different from previous reviews. We will use a larger group of articles for analysis than the former review [15], and it will analyze effectiveness, different from the latter review [9].

## 2. Methods

### 2.1. Eligibility Criteria

Articles eligible for this review required older adults (>50) with early-stage Alzheimer’s Disease or MCI as participants, published in the last ten years, published in peer-reviewed journals, and used strong methods such as RCT or true experiments. Other methods were accepted such as quasi-experimental, mixed method, quantitative, and qualitative.

### 2.2. Information Sources

We searched in four well-known databases: PubMed (MEDLINE), Complete Index of Nursing and Allied Health Literature (CINAHL), Web of Science, and Embase’s ScienceDirect. We conducted the search on 3 August 2022. We also performed a journal-specific search of Healthcare. MEDLINE was excluded from all but PubMed. We eliminated reviews from our search to not confound the results. We used only published literature to ensure it was peer reviewed.

### 2.3. Search Strategy

We visited the U.S. Library of Medicine’s website to use the Medical Subject Heading’s (MeSH) indexing database. Using MeSH, we created a Boolean search string to combine key terms. We used the same search sting in all databases: (mhealth OR telemedicine OR “virtual reality” OR “serious games”) AND (“Alzheimer disease” OR dementia) AND memory. Due to differences in filter options in each database, we could not use the exact same filters, but we used similar filter strategies. In CINAHL, we filtered by date, full-text, humans, English language, academic journals, excluded MEDLINE, and excluded reviews. In ScienceDirect, we filtered by date, excluded MEDLINE, and excluded reviews and conference proceedings. In Web of Science, we filtered by date, excluded reviews, and excluded MEDLINE. This practice eliminated most duplicates.

### 2.4. Selection Process

In accordance with the Kruse Protocol, we searched key terms in all databases, filtered results, and screened abstracts for applicability [16]. At least two reviewers screened each abstract, and at least two reviewers analyzed each article for data extraction and thematic analysis.

### 2.5. Data Collection Process

The Kruse Protocol standardized an Excel spreadsheet for data extraction and analysis. We used a series of three consensus meetings to finalize the group of articles for analysis, identify themes in the literature, and perform additional analysis on the data extracted.

### 2.6. Data Items

In accordance with the Kruse Protocol, we collected the following fields of data: database source, date of publication, authors, title of study, participant population, experimental intervention, results (compared to a control), medical outcomes, sample size, bias within study, effect size (Cohen’s *d*), sensitivity, specificity, F1, country of origin, statistics used, patient satisfaction, effectiveness, barriers to adoption, strength of evidence, and quality of evidence. Results were reported in comparison to a control group. Outcomes and effectiveness are highly similar fields, but they are designed for different audiences (providers and administrators). A provider might not be as concerned as length of stay or cost savings as much as direct medical outcomes (e.g., improvement in cognition), but the administrator is.

The primary outcome for this study is cognition, as measured by the MMSE or similar tool such as Addenbrooke’s Cognitive Examination-Revised (ACE-R), Cognitive Failures Questionnaire (CFQ), Wechsler Adult Intelligence Scale (WAIS), or Alzheimer’s Disease Assessment Scale-cognitive subscale (ADAS-Cog). Secondary outcomes are reported by studies through a range of measurement tools such as story recall, Hamilton Depression Rating Scale (HAMD), Wechsler Memory Scale 3rd edition (WMS-III), Rey-Osterrieth Complex Figure (ROCF), Controlled Oral Words Association Test (COWAT), Symbol Digit Modalities Test (SDMT), Bayer Activities of Daily Living, etc.

### 2.7. Study Risk of Bias Assessment and Reporting Bias Assessment

Not only did reviewers note observations of bias in each study, but we also assessed the strength and quality of each study using the Johns Hopkins Nursing Evidence Based Practice tool (JHNEBP) [17]. The overall ratings of quality from the JHNEDP provided us with an assessment of the applicability of the cumulative evidence.We considered the instances of bias in how to interpret the results because bias can limit external validity [18].

### 2.8. Effect Measures

Because we accepted mixed methods and qualitative studies, we were unable to standardize summary measures, as would be performed in a meta-analysis. Measures of effect are summarized in tables for those studies in which it was reported. Measures of effect can be reported as Cohen’s *d*, Wald’s *W*, *Eta*^2^, sensitivity, or specificity. Effects vary based on the statistic used, but they usually follow small (0.0–0.2), medium (0.21–0.79), large (0.8 or higher). An average effect size (ES) can be calculated through a weighted average by using the sample size.

### 2.9. Synthesis Methods

We performed a thematic analysis of the data combining observations (observed multiple times) into themes [19]. We calculated the frequency of occurrences and reported the findings in a series of affinity matrices. This frequency reporting states the probability of finding that theme in the group for analysis, and it provides confidence in the data analyzed. Although thematic analyses are usually reserved for qualitative studies, there is a pattern in the literature for systematic literature reviews to utilize this technique to help synthesize data extracted [20,21,22].

### 2.10. Additional Analyses and Certainty Assessment

Using the standardized spreadsheet, we sorted by intervention and theme to identify interactions. Some interventions appear more effective than others. Sensitivity and specificity were tabulated where reported.

## 3. Results

### 3.1. Study Selection

**Figure 1:**
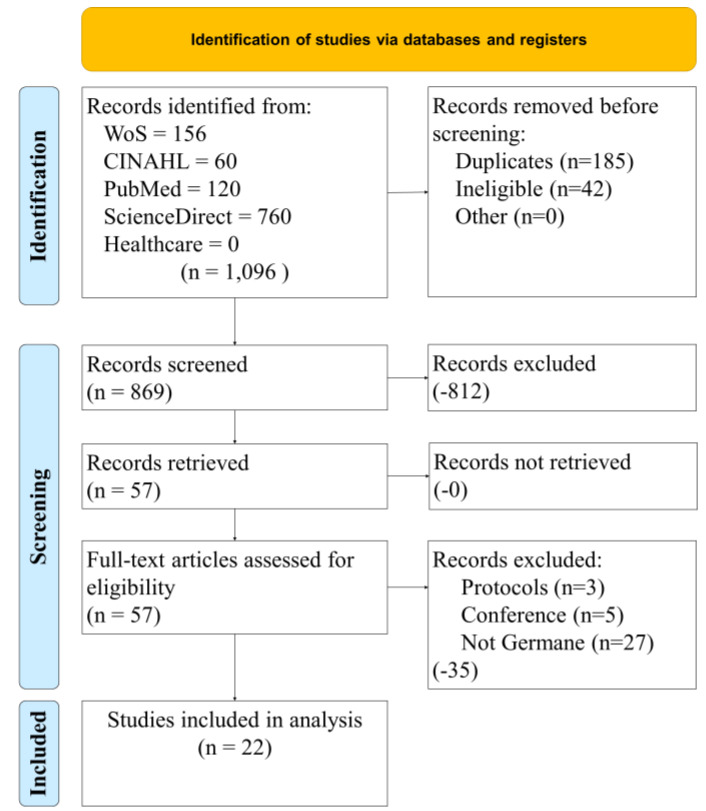
Study selection process.

Figure 1 illustrates our study selection process. Four databases and one focused journal search were conducted with a standardized Boolean search string. The initial 1096 results were filtered to remove duplicates. At the end of the filtering exercise, 869 records were screened using filters on each database. This exercise removed 812 articles. The resulting 57 were retrieved for a full analysis for eligibility. Several more were filtered out (protocols, conference papers, and those that were not germane to our research objective). The remaining group for analysis was 22.

### 3.2. Study Characteristics

Following the PRISMA 2020 checklist, characteristics for each study were systematically extracted and tabulated to include the following data fields: participants, intervention, comparison (to control or other group), observation, study design (PICOS). The standard PICOS table summarizes study characteristics in a manner commensurate with the literature (See Table 1). Of the 22 studies analyzed over the 10-year period, 0 were from 2012, 1 was from 2013 [23], 3 were from 2014 [24,25,26], 2 were from 2015 [27,28], 4 were from 2016 [29,30,31,32], 2 were from 2017 [33,34], 2 were from 2018 [35,36], 3 were from 2019 [37,38,39], 3 were from 2020 [40,41,42], 2 were from 2021 [43,44], and 0 were from 2022. All studies involved older adults mostly above 50 years except one study where participants with MCI were above 42 years. The interventions were heavily loaded with mHealth and eHealth (13/22, 59%), while 6/22 (27%) were VR, and 3 were a combination of telephone, mHealth + VR, and a game console. About 73% (16/22) of the studies were RCTs, 2 were either quasi-experimental or pre-post (using a control), and one each for observational and mixed-methods. Of the 16 RCTs, only 5 provided effect sizes (ES). The weighted average ES was 1.48. Studies originated in 13 different countries, but half were from Korea, the United States, and Italy.

### 3.3. Risk of Bias in and across Studies

Reviewers exercised the JHNEBP quality assessment tool to identify strength and quality of evidence. Reviewers also made notes of other observations of bias throughout the data extraction. The JHNEBP tool identified 16/22 (73%) of Strength I due to the use of strong methodologies such as RCT and true experiment. Four others (18%) were identified as Strength II due to either quasi-experimental or a pre-post with a control group. Only 2/22 (9%) were identified as Strength III because of the use of observational or mixed methods methodologies. The JHNEBP tool also identified 16/22 (73%) as Quality A due to the use of adequate control groups and sample sizes, and for reporting consistent results. Only 6/22 (27%) were identified as Quality B. No studies were identified as less than Strength III or Quality B.

Reviewers also identified other incidents of bias. [18] There were 22 observations of selection bias, which threatens the internal validity of the studies. These observations stemmed from limiting the population to one region or one country. Reviewers also noted four observations of sample bias, which threatens the external validity of the studies. These observations were noted where the population was a majority of one race or gender. There were two observations of design bias, which threatens the internal validity of the study. These were noted when there seemed to be a significant flaw in the methodology (e.g., short intervention time).

### 3.4. Results of Individual Studies

Table 2 summarized the results of individual studies. This table shows the themes identified in the literature. In multiple occasions, there were multiple observations of the same theme identified in the same study. This was an artifact of collapsing observations of a similar nature into one theme. An observation-to-theme match can be found in Appendix A. Other observations incident to the data extraction can be found in Appendix B (sample size, bias, effect size, country of origin, statistics used, patient satisfaction, and the JHNEBP strength and quality of evidence).

### 3.5. Results of Syntheses, Additional Analysis, and Certainty of Evidence

We conducted a thematic analysis of the literature to make sense of the data extracted. Through this process, observations noted multiple times became themes. Not all observations were fit into themes: Some remained as individual observations. These themes and observations are reported by category in affinity matrices with frequency distributions. Frequencies do not imply importance—instead they identify the probability the theme was identified in the group of articles analyzed.

#### 3.5.1. Patient Satisfaction

Observations of patient satisfaction can be found in Appendix C. This appendix tabulates the. Only two themes and two individual observations were made. Patients commented their appreciation and how they valued the technology inherent to the interventions. This theme appeared in 11/32 (34%) of the observations [23,26,28,29,30,31,32,33,34,35,36]. The interventions had a positive effect on the patient experience. This appeared in 10/30 (32%) of the observations [23,24,26,27,28,29,30,31,33,34]. The intervention improved cognitive function in one study [25], and the technology frustrated patients in another study [37].

#### 3.5.2. Results to the Adoption of mHealth and VR for Memory Care for AD Patients

Table 3 summarizes the results incident to the intervention of mHealth and VR for memory care. Six themes and seven individual observations were identified by the reviewers for a total of 41 occurrences in the literature. Nine interventions improved cognition, as measured by the MMSE, ADAS-Cog, or WAIS tests [24,25,26,29,32,34,35,38,43]. Seven interventions improved memory [23,28,30,31,34,36,40]. Five interventions improved language [23,24,25,31,34]. Four interventions improved brain activity, as measured by EEG [33,38,40,42]. Four interventions improved attention [31,34,36,41], and three improved vitality [31,36,40]. One intervention improved cortical atrophy [23]. One intervention improved resistance training through a combination of resistance and cognitive training protocol [25]. One intervention improved both quality of life and mental health [36]. One intervention improved both cerebral blood flow and neuro plasticity [38]. One intervention improved depression [27]. Only three interventions showed either no improvements or no significant improvements [37,39,44].

#### 3.5.3. Medical Outcome Commensurate with the Adoption of mHealth and VR for Memory Care

Table 4 summarizes the medical outcomes observed. Six themes and seven individual observations were recorded commensurate with the adoption of mHealth and VR for memory care for patients with AD, for a total of 41 occurrences. The results and medical outcomes are highly similar.

#### 3.5.4. Effectiveness Themes and Observations

Table 5 summarizes the medical outcomes observed. Six themes and seven individual observations were recorded commensurate with the adoption of mHealth and VR for memory care for patients with AD, for a total of 41 occurrences. The medical outcomes and Effectiveness themes are highly similar. The only difference was that two interventions noted a time savings by using the intervention [34,35].

#### 3.5.5. Barriers to the Adoption of mHealth and VR for Memory Care for Patients with AD

Table 6 summarizes the barriers to the adoption of mHealth and VR for memory care for patients with AD. Four themes and one individual observation was recorded commensurate with the adoption of the interventions, for a total of 88 occurrences. The most common barriers, which occurred together in many of the studies, was time of providers (to manage the intervention and administer tests) [23,24,25,26,27,28,29,30,31,32,33,34,35,36,37,38,39,40,41,42,43,44], training (providers, staff, and patients) [23,24,25,26,27,28,29,30,31,32,33,34,35,36,37,38,39,40,41,42,43,44], cost (of technology and tests) [23,25,26,27,28,29,30,31,32,33,34,35,36,37,38,39,40,41,42,43,44], low reimbursement (which is highly correlated with cost) [23,25,26,27,28,29,30,31,32,33,34,35,36,37,38,39,40,41,42,43,44], and dexterity limitations of older adults [33].

#### 3.5.6. Interactions between Observations

About 60% of the interventions were mHealth, eHealth. This intervention was associated with improvements in cognition [25,29,32,34,35], memory [23,30,31,34,36], language [23,25,31,34], attention [31,34,36,41], brain activity [33], cortical atrophy [23], resistance training [25], and depression [27]. Only one study that used this intervention reported no improvement [37]. The VR interventions reported improved cognition [26,43], brain activity [40,42], memory [40], and vitality [40]. Two VR studies reported either no improvement or no statistically significant improvements [39,44]. The mHealth + VR intervention reported improved memory [28]. The game console intervention reported improved cognition, brain activity, cerebral blood flow, and neuro plasticity [38]. The telephone intervention reported an increase in cognition and language [24].

## 4. Discussion

### 4.1. Summary of Evidence

This systematic literature review analyzed 22 studies from 13 countries published over 10 years to analyze the effectiveness of mHealth and VR for memory care for patients with AD. Five interventions were identified; however, the dominant intervention was mHealth, eHealth. The lines between mHealth and eHealth are significantly blurred due to the capabilities of mobile devices. This intervention comprised 13/22 (59%) of the studies. Virtual Reality was the most often cited intervention, appearing in 6/22 (27%) studies. Methodologies were very strong in the studies analyzed. About 73% of the studies used RCT as the study design [23,25,26,28,29,30,31,34,35,36,37,38,39,41,42,43]. The strong study designs resulted in a low rate of bias within and among studies because the studies used adequate sample sizes and controls, and they reported consistent results. Very small observations of internal and external bias were observed in all studies. There were 9 instances of an improvement of cognition [24,25,26,29,32,34,35,38,43], 7 instances of an improvement in memory [23,28,30,31,34,36,40], 5 instances of an improvement in language [23,24,25,31,34], four improvements in EEG scores [33,38,40,42], four improvements in attention [31,34,36,41] three improvements in vitality [31,36,40], and several individual improvements in cortical atrophy, resistance training, quality of life, mental health, cerebral blood flow, depression, and neuro plasticity [25,27,36,38].

This review highlights are large diversity of results from these five interventions. The mHealth and eHealth interventions consistently showed the largest improvements in cognition [25,29,32,34,35], memory [23,30,31,34,36], language [23,25,31,34], attention [31,34,36,41], brain activity [33], cortical atrophy [23], resistance training [25], and depression [27]. The game console intervention reported improvements in several areas: cognition, brain activity, cerebral blood flow, and neuro plasticity [38]. The VR interventions did not report as many improvements: cognition [26,43], brain activity [40,42], memory [40], and vitality [40]. The telephone intervention reported improvements in two areas: cognition and language [24]. The mHealth + VR intervention only improved memory [28].

Future research should focus on the improvements in cognition, memory, and brain waves to identify the duration of the improvements. The studies analyzed did not imply the results would be long term. Both mHealth and VR offer some good interventions to provide temporal relief and improvement of AD symptoms. Only three studies identified no improvement or no statically significant improvement [37,39,44]. The rest identified improvements in at least one area. Future considerations should focus on the interventions with the largest reported improvements. In this review, those would be mHealth, eHealth.

The results of this review should provide options for providers and care givers who want to see an improvement in one area or another. The results of these studies are positive. However, providers do face several barriers to the adoption of these interventions. The cost to acquire the equipment would not currently be reimbursed with current treatment codes. It would help to codify some of these interventions into critical practice guidelines. An existing CPG would have a better chance of being reimbursed. After acquiring the equipment, the provider would need to train the staff and the users of the equipment for each intervention. The provider and staff would need additional time to operate the equipment, administer and analyze the measurement tests like the MMSE, and EEG. These barriers are not compelling, but they present significant stumbling blocks to universal adoption.

### 4.2. Limitations

To control for sample bias, we queried four well-known databases, and we used every article that emerged from the abstract screening step. We chose only four databases, but others may have identified additional studies with additional interventions. We also limited the search to published articles that had been peer reviewed. This publication bias may have prevented us from identifying other interventions with various margins of success. To control for confirmation bias, we had multiple reviewers participate in every step: screening, data extraction, and analysis. To control for design bias, we stuck with a published protocol aligned with more than 40 published systematic literature reviews.

### 4.3. Conclusions

mHealth and VR offer promising interventions to help memory and cognition for those who suffer from AD. Several interventions show temporary improvement in cognition, memory, and brain activity. The mHealth and eHealth interventions seem to affect a larger scope of measurable criteria, and they may be easier to implement without complicated VR apparatus. Several barriers stand in the way of universal adoption. Additional reimbursement mechanisms would enable providers to adopt these interventions or test them under different circumstances. The AD patients and their caregivers look for answers and an improvement in the AD symptoms. With additional development, mHealth and VR might provide some viable solutions.

## Figures and Tables

**Table 1:** PICOS.

Authors	Participants	Experimental Intervention	Results (Compared to Control Group)	Medical Outcomes Reported	Study Design
Zhuang et al. [23]	Older Adult (≥70), average age 83, 24% male, 76% female, all Asian (Chinese)	mHealth, eHealth cognitive training program	Intervention group with global cortical atrophy (GCA) showed improvement (*p* < 0.05). No change with baseline cognitive exam.	Improvement in memory, language, and visuospatial abilities	RCT
Jelcic et al. [24]	Older Adult (≥80), average age 83, 22% male, 77% female, 100% Caucasian	Telephone-based	The mean Mini Mental State Examination (MMSE) scores improved significantly in telecommunication technology (LSS-tele) and LSS-direct treatments	Improvement in working memory and semantic fluency	Quasi-experimental
Singh et al. [25]	Older Adult (>55), average age 68.5, 68% female	mHealth, eHealth multidomain cognitive training	Resistance training was 74% higher for executive domain compared with combined training, cognition, and verbal memory	improvement in global cognition, executive function and verbal/constructional memory	RCT
Tarnanas et al. [26]	Older Adult (>65), average age 70.5, 73% male,77% Caucasian	Virtual Reality (VR), and Augmented Reality (AR)	improvements of specific cognitive functions and working memory	improves untrained cognitive functions in MCI	RCT
Burdea et al. [27]	Adults (>50 years) with MCI, 70% male	mHealth (BrightBrainer) app	(*p* < 0.05)Improvement in decision making, with trend improvements in depression. Non-statistically significant results found in processing speed and auditory attention.	Improvements in decision making and depression	Pre-post
Finn et al. [28]	Older Adult (>65), average age 75, 71% male, 29% female, 100% Caucasian	mHealth, VR, Telemedicine	(*p* < 0.05)- Improved task performance over the course of training.	Repetition-lag training (RLT), a form of recognition memory training reported	RCT
Callan et al. [29]	Older Adult (>64), average age 75, 100% Caucasian, non Latino	mHealth cognitive training task (APVSAT)	Improved task performance, in terms of speed, by nearly 50%	Reported as useful approach for incorporating device usage into daily routines.	RCT
Cavallo et al. [30]	Older Adult (>75), average age 76, 100% Caucasian	Structured rehabilitative software	(*p* < 0.05)-improvement in the intervention group greater than the control.	Improvement in memory	RCT
Hagovska et al. [31]	Older Adult(≥65 years of age), average age 67.07, female 51.02% male 49% male, 100% Caucasian	Training battery prog- Cogni-Plus, SCHUHFRIED GmbH Austria, Dynamic balance training	(*p* < 0.05) improvement in postural reactions, attention, memory and language ability in the intervention group	Improvement in postural reactions, attention, memory and language	RCT
Hyer et al. [32].	Older Adult (≥65 years) average age 75, female 53% male 47%, 89% white, 11% black	Cogmed or a Sham computer program. For Repeatable Battery for Neuropsychological Status and the Clinical Dementia Rating	Cogmed group demonstrated better performance on the Functional Activities Questionnaire (FAQ), a measure of adjustment and far transfer, at follow-up.	Both groups, especially Cogmed, enjoyed the intervention. Cognitive stimulation activities improved mental skills	Pre-post
Boyd et al. [33]	Older Adult (≥74 years) average age 78, female 68% male 31%, Caucasian	Trials to use Apps-evaluation of EnCare diagnostics (ECD) and the brain fit plan (BFP) in healthy older adults	No control group. Improved brain waves	ECD is highly acceptable in both healthy older adults and those with early-stage dementia when given the shorter versions to accommodate their diagnosis.	Observational
Yang et al. [34]	Older Adult (≥68 years) average age 70, female 68% male 31%, Caucasian	24 sessions of computer-based cognitive training, over a 12 week period.	Computer-based cognitive treatment resulting in self-training and self-learning of a patient	Improvement in language, attention, calculation, verbal memory, and frontal function for the experimental group	RCT
Lee et al. [35]	Older Adult (≥70 years) average age 74.3, female 60% male 40%	12 sessions of a computerized cognitive rehabilitation program for three weeks	“No control group”. Two treatment groups only	Improved attention in subjects who underwent computerized cognitive rehabilitation using Bettercog.	RCT
Park et al. [36]	Older Adult (≥60 years) average age 66.5, female 47% male 53%	NCT group showed improvement in vitality, role-emotional, and mental health compared with the CCT group	Cognitive function (attention, memory, and visual spatial ability) showed a significant increase in both groups *p* < 0.05), as did the mental components of health-related quality of life (*p* < 0.05)	Regarding health-related quality of life, the NCT group showed more improvement in vitality, role-emotional, and mental health compared with the CCT group	RCT
Flak et al. [37]	Adults (>42 years) with MCI, 66% male	mHealth memory training app	Adaptive training group did not show significantly greater improvement on the main outcome of working memory performance at 1 and 4 months after training	no improvement	RCT
Kahn [38]	Adults (>50 years) with MCI	game console with cognitive games	Theta, delta waves and complexity of EEG significantly improved	Xbox 360 Kinect cognitive games improved EEG indicators and cognitive functions, and, 15–17 increasing cerebral blood flow,59 neural plasticity,60 activation of arousal system,61 neurotransmitters modulation	RCT
Park [39]	Adults (>50 years) with MCI	culture based virtual reality	VR-based training group exhibited no significant differences following the three-month VR program	no improvement	RCT
Park et al. [40]	Adults (>59 years, avg age 70.4), with MCI	VR	No control group. improvement in physical, memory and brain stimulation, but the participants have a low focus on decision making	Improvement in physical outcomes, memory and brain stimulation	Mixed Methods
Robert et al. [41]	Adults (>50 years, avg age 79.4), with MCI	mHealth app (MeMo)	Significant differences in two attention tests	significant differences in two attention tests	RCT
Thapa et al. [42]	Adults (>50 years) with MCI	VR	Intervention group exhibited a significantly improved executive function and brain function at the resting state	Intervention group exhibited a significantly improved executive function and brain function at the resting state	RCT
Oliveria et al. [43]	Adults (>50 years) with MCI	VR	Improvement in overall cognitive function in the experimental group	Improvement in overall cognitive function in the experimental group	RCT
Seredakis et al. [44]	Adults (>50 years) with MCI	VR	No group interaction	No group interaction	Quasi-experimental

**Table 2:** Summary of analysis, sorted chronologically.

Authors	Intervention Themes	Results Themes	Outcome Themes	Effectiveness Themes	Barrier Themes
Zhuang et al. [23]	mHealth, eHealth	Improvement in cortical atrophy	Improvement in cortical atrophy	Improvement in cortical atrophy	Cost
		Improved memory	Improvedmemory	Improved memory	Training
		Improved language	Improved language	Improved language	Low reimbursement
					Time of providers
Jelcic et al. [24]	Telephone	Improved MMSE scores (cognition)	Improved MMSE scores (cognition)	Improved MMSE scores (cognition)	Time of providers
		Improved language	Improved language	Improved language	Training
					Time of providers
Singh et al. [25]	mHealth, eHealth	Improved resistance training	Improved resistance training	Improved resistance training	Cost
		Improved ADAS-Cog scores (cognition)	Improved ADAS-Cog scores (cognition)	Improved ADAS-Cog scores (cognition)	Training
		Improved language	Improved language	Improved language	Low reimbursement
					Time of providers
Tarnanas et al. [26]	Virtual Reality (VR)	Improved MMSE scores (cognition)	Improved MMSE scores (cognition)	Improved MMSE scores (cognition)	Cost
					Training
					Low reimbursement
					Time of providers
Burdea et al. [27]	mHealth, eHealth	Improved depression	Improved depression	Improved depression	Cost
					Training
					Low reimbursement
					Time of providers
Finn et al. [28]	mHealth + VR	Improved memory	Improved memory	Improved memory	Cost
					Training
					Low reimbursement
					Time of providers
Callan et al. [29]	mHealth, eHealth	Improved MMSE scores (cognition)	Improved MMSE scores (cognition)	Improved MMSE scores (cognition)	Cost
					Training
					Low reimbursement
					Time of providers
Cavallo et al. [30]	mHealth, eHealth	Improved memory	Improved memory	Improved memory	Cost
					Training
					Low reimbursement
					Time of providers
Hagovska et al. [31]	mHealth, eHealth	Improved attention	Improved attention	Improved attention	Cost
		Improved memory	Improved memory	Improved memory	Training
		Improved language	Improved language	Improved attention	Low reimbursement
		Improved vitality	Improved vitality	Improved language	Time of providers
Hyer et al. [32]	mHealth, eHealth	Improved CFQ scores (cognition)	Improved CFQ scores (cognition)	Improved CFQ scores (cognition)	Cost
					Training
					Low reimbursement
					Time of providers
Boyd et al. [33]	mHealth, eHealth	Improved EEG scores (brain waves)	Improved EEG scores (brain waves)	Improved EEG scores (brain waves)	Dexterity limitations of older adults
					Cost
					Training
					Low reimbursement
					Time of providers
Yang et al. [34]	mHealth, eHealth	Improved K-MMSE scores (cognition)	Improved K-MMSE scores (cognition)	Improved K-MMSE scores (cognition)	Cost
		Improved memory	Improved memory	Improved memory	Training
		Improved language	Improved language	Improved language	Low reimbursement
		Improved attention	Improved attention	Savings in time	Time of providers
				Improved attention	
Lee et al. [35]	mHealth, eHealth	Improved MMSE scores (cognition)	Improved MMSE scores (cognition)	Savings in time	Cost
				Improved MMSE scores (cognition)	Training
					Low reimbursement
					Time of providers
Park et al. [36]	mHealth, eHealth	Improved attention	Improved attention	Improved attention	Cost
		Improved memory	Improved memory	Improved MMSE scores (memory)	Training
		Improved vitality	Improved vitality	Improved vitality	Low reimbursement
		Improved mental health	Improved mental health	Improved mental health	Time of providers
		Improved quality of life	Improved quality of life	Improved quality of life	
Flak et al. [37]	mHealth, eHealth	No improvement	None reported	None reported	Cost
					Training
					Low reimbursement
					Time of providers
Kahn [38]	Game console	Improved EEG scores (brain waves)	Improved EEG scores (brain waves)	Improved EEG scores (brain waves)	Cost
		Improved MMSE scores (cognition)	Improved MMSE scores (cognition)	Improved MMSE scores (cognition)	Training
		Improved cerebral blood flow	Improved cerebral blood flow	Improved cerebral blood flow	Low reimbursement
		Improved neuro plasticity	Improved neuro plasticity	Improved neuro plasticity	Time of providers
Park [39]	Virtual Reality (VR)	No significant differences	None reported	None reported	Cost
					Training
					Low reimbursement
					Time of providers
Park et al. [40]	Virtual Reality (VR)	Improved vitality	Improved vitality	Improved vitality	Cost
		Improved memory	Improved memory	Improved memory	Training
		Improved EEG scores (brain waves)	Improved EEG scores (brain waves)	Improved EEG scores (brain waves)	Low reimbursement
					Time of providers
Robert et al. [41]	mHealth, eHealth	Improved attention	Improved attention	Improved attention	Cost
					Training
					Low reimbursement
					Time of providers
Thapa et al. [42]	Virtual Reality (VR)	Improved EEG scores (brain waves)	Improved EEG scores (brain waves)	Improved EEG scores (brain waves)	Cost
					Training
					Low reimbursement
					Time of providers
Oliveria et al. [43]	Virtual Reality (VR)	Improved MMSE scores (cognition)	Improved MMSE scores (cognition)	Improved MMSE scores (cognition)	Cost
					Training
					Low reimbursement
					Time of providers
Seredakis et al. [44]	Virtual Reality (VR)	No improvement	None reported	None reported	Cost
					Training
					Low reimbursement
					Time of providers

**Table 3:** Results to the adoption of mHealth and VR for memory care.

Results Themes and Observations	Frequency
Improved cognition (MMSE, ADAS-Cog, WAIS) [24,25,26,29,32,34,35,38,43]	9
Improved memory [23,28,30,31,34,36,40]	7
Improved language [23,24,25,31,34]	5
Improved EEG scores (brain waves) [33,38,40,42]	4
Improved attention [31,34,36,41]	4
Improved vitality [31,36,40]	3
No improvement [37,44]	2
Improvement in cortical atrophy [23]	1
Improved resistance training [25]	1
Improved quality of life [36]	1
Improved mental health [36]	1
Improved cerebral blood flow [38]	1
Improved depression [27]	1
No significant differences [39]	1
Improved neuro plasticity [38]	1
	41

**Table 4:** Medical outcomes commensurate with the adoption of mHealth and VR.

Outcomes Themes and Observations	Frequency
Improved cognition (MMSE, ADAS-Cog, WAIS) [24,25,26,29,32,34,35,38,43]	9
Improved memory [23,28,30,31,34,36,40]	7
Improved language [23,24,25,31,34]	5
Improved EEG scores (brain waves) [33,38,40,42]	4
Improved attention [31,34,36,41]	4
Improved vitality [31,36,40]	3
None reported [37,39,44]	3
Improvement in cortical atrophy [23]	1
Improved resistance training [25]	1
Improved quality of life [36]	1
Improved mental health [36]	1
Improved cerebral blood flow [38]	1
Improved neuro plasticity [38]	1
Improved depression [27]	1
	41

**Table 5:** Effectiveness of mHealth and VR for memory care for patients with AD.

Effectiveness Themes and Observations	Frequency
Improved MMSE scores (cognition) [24,25,26,29,32,34,35,38,43]	9
Improved MMSE scores (memory) [23,28,30,31,34,36,40]	7
Improved language [23,24,25,31,34]	5
Improved attention [31,34,36,41]	4
Improved EEG scores (brain waves) [33,38,40,42]	4
Improved vitality [31,36,40]	3
None reported [37,39,44]	3
Savings in time [34,35]	2
Improvement in cortical atrophy [23]	1
Improved resistance training [25]	1
Improved quality of life [36]	1
Improved mental health [36]	1
Improved cerebral blood flow [38]	1
Improved neuro plasticity [38]	1
Improved depression [27]	1
	43

**Table 6:** Barriers to the adoption of mHealth and VR for memory care.

Barrier Themes and Observation	Frequency
Time of providers [23,24,25,26,27,28,29,30,31,32,33,34,35,36,37,38,39,40,41,42,43,44] *	23
Training [23,24,25,26,27,28,29,30,31,32,33,34,35,36,37,38,39,40,41,42,43,44]	22
Cost [23,25,26,27,28,29,30,31,32,33,34,35,36,37,38,39,40,41,42,43,44]	21
Low reimbursement [23,25,26,27,28,29,30,31,32,33,34,35,36,37,38,39,40,41,42,43,44]	21
Dexterity limitations of older adults [33]	1
	88

* Multiple occurrences in one study.

## Data Availability

Data from this study can be obtained by asking the lead author.

## Protocol and Registration

This review was conducted in accordance with the Kruse Protocol for writing a systematic review. It was reported in accordance with the Preferred Reporting Items for Systematic Reviews and Meta-Analysis (PRISMA). This review is registered with PROSPERO: CRD42021266730.

## Appendix A

**Table A1:** Observation-to-theme conversion (Intervention, Results, and Medical Outcomes).

Authors	Experimental Intervention	Intervention Themes	Results (Compared to Control Group)	Results Themes	Medical Outcomes Reported	Outcome Themes
Zhuang et al.	mHealth, eHealth cognitive training program	mHealth, eHealth	Intervention group with global cortical atrophy (GCA) showed improvement (*p* < 0.05). No change with baseline cognitive exam.	Improvement in cortical atrophy	Improvement in memory, language, and visuospatial abilities	Improvement in cortical atrophy
				Improved memory		Improved memory
				Improved language		Improved language
Jelcic et al.	Telephone-based	Telephone	The mean Mini Mental State Examination (MMSE) score improved significantly in Telecommunication technology (LSS-tele) and LSS-direct treatments	Improved MMSE scores (cognition)	Improvement in working memory and semantic fluency	Improved MMSE scores (cognition)
				Improved language		Improved language
Singh et al.	mHealth, eHealth multidomain cognitive training	mHealth, eHealth	Resistance training was 74% higher for Executive Domain compared with combined training, cognition, and verbal memory	Improved resistance training	Improvement in global cognition, executive function and verbal/constructional memory	Improved resistance training
				Improved MMSE scores (cognition)		Improved MMSE scores (cognition)
				Improved language		Improved language
Tarnanas et al.	Virtual Reality (VR), and Augmented Reality (AR)	Virtual Reality (VR)	improvements of specific cognitive functions and working memory	Improved MMSE scores (cognition)	improves untrained cognitive functions in MCI	Improved MMSE scores (cognition)
Burdea et al.	mHealth (BrightBrainer) app	mHealth, eHealth	statistically significant improvement in decision making, with trend improvements in depression. Non-statistically significant results found in processing speed and auditory attention.	Improved depression	Improvements in decision making and depression	Improved depression
Finn et al.	mHealth, VR, Telemedicine	mHealth + VR	(*p* < 0.05). Improved on the task itself over the course of training.	Improved memory	repetition-lag training (RLT), a form of recognition memory training reported	Improved memory
Callan et al.	mHealth cognitive training task (APVSAT)	mHealth, eHealth	Improved task performance, in terms of speed, by nearly 50%	Improved MMSE scores (cognition)	Reported as useful approach for incorporating device usage into daily routines.	Improved MMSE scores (cognition)
Cavallo et al.	structured rehabilitative software	mHealth, eHealth	(*p* < 0.05). Improvement in the intervention group greater than the control.	Improved memory	Improvement in memory	Improved memory
Hagovska et al.	Training battery prog- Cogni-Plus, SCHUHFRIED GmbH Austria, Dynamic balance training	mHealth, eHealth	(*p* < 0.05). improvement in postural reactions, attention, memory and language ability in the intervention group	Improved attention	improvement in postural reactions, attention, memory and language	Improved attention
				Improved memory		Improved memory
				Improved language		Improved language
				Improved vitality		Improved vitality
Hyer et al.	Cogmed or a Sham computer program. For Repeatable Battery for Neuropsychological Status and the Clinical Dementia Rating	mHealth, eHealth	The Cogmed group demonstrated better performance on the Functional Activities Questionnaire (FAQ), a measure of adjustment and far transfer, at follow-up.	Improved MMSE scores (cognition)	Both groups, especially Cogmed, enjoyed the intervention. Cognitive stimulation activities improved mental skills	Improved MMSE scores (cognition)
Boyd et al.	Trials to use Apps-evaluation of EnCare diagnostics (ECD) and the brain fit plan (BFP) in healthy older adults	mHealth, eHealth	No control group. Improved brain waves	Improved EEG scores (brain waves)	ECD is highly acceptable in both healthy older adults and those with early stage dementia when given the shorter versions to accommodate their diagnosis.	Improved EEG scores (brain waves)
Yang et al.	24 sessions of computer-based cognitive training, over a 12 week period.	mHealth, eHealth	Computer-based cognitive treatment resulting in self-training and self-learning of a patient	Improved MMSE scores (cognition)	Improvement in language, attention, calculation, verbal memory, and frontal function for the experimental group	Improved MMSE scores (cognition)
				Improved memory		Improved memory
				Improved language		Improved language
Lee et al.	12 sessions of a computerized cognitive rehabilitation program for three weeks	mHealth, eHealth	“No control group”. Two treatment groups only	Improved MMSE scores (cognition)	Improvement in subjects who underwent computerized cognitive rehabilitation using Bettercog.	Improved MMSE scores (cognition)
Park et al.	NCT group showed improvement in vitality, role-emotional, and mental health compared with the CCT group	mHealth, eHealth	Cognitive function (attention, memory, and visual spatial ability) showed a significant increase in both groups (*p* < 0.05), as did the mental components of health-related quality of life (*p* < 0.05)	Improved attention	Regarding health-related quality of life, the NCT group showed more improvement in vitality, role-emotional, and mental health compared with the CCT group	Improved attention
				Improved memory		Improved memory
				Improved vitality		Improved vitality
				Improved mental health		Improved mental health
				Improved quality of life		Improved quality of life
Flak et al.	mHealth memory training app	mHealth, eHealth	Adaptive training group did not show significantly greater improvement on the main outcome of working memory performance at 1 and 4 months after training	No improvement	no improvement	None reported
Kahn	Game console with cognitive games	Game console	Theta, delta waves and complexity of EEG significantly improved	Improved EEG scores (brain waves)	Xbox 360 Kinect cognitive games improved EEG indicators and cognitive functions probably through multiple mechanisms, such as, cognition improvement, 15–17 increasing cerebral blood flow, 59 neural plasticity, 60 activation of arousal system, 61 neurotransmitters modulation	Improved EEG scores (brain waves)
				Improved MMSE scores (cognition)		Improved MMSE scores (cognition)
				Improved cerebral blood flow		Improved cerebral blood flow
				Improved neuro plasticity		Improved neuro plasticity
Park	culture based virtual reality	Virtual Reality (VR)	VR-based training group exhibited no significant differences following the three-month VR program	No significant differences	no significant improvements noted	None reported
Park et al.	VR	Virtual Reality (VR)	No control group. improvement in physical, memory and brain stimulation, but the participants have a low focus on decision making	Improved vitality	Improvement in physical outcomes, memory and brain stimulation	Improved vitality
				Improved memory		Improved memory
				Improved EEG scores (brain waves)		Improved EEG scores (brain waves)
Robert et al.	mHealth app (MeMo)	mHealth, eHealth	Significant differences in two attention tests	Improved attention	Improvement in attention tests	Improved attention
Thapa et al.	VR	Virtual Reality (VR)	Intervention group exhibited a significantly improved executive function and brain function at the resting state	Improved EEG scores (brain waves)	Intervention group exhibited a significantly improved executive function and brain function at the resting state	Improved EEG scores (brain waves)
Oliveria et al.	VR	Virtual Reality (VR)	an improvement in overall cognitive function in the experimental group	Improved MMSE scores (cognition)	an improvement in overall cognitive function in the experimental group	Improved MMSE scores (cognition)
Seredakis et al.	VR	Virtual Reality (VR)	No group interaction	No improvement	No group interaction	None reported

## Appendix B

**Table A2:** Observation-to-theme conversion (Effectiveness and Barriers to adoption).

Authors	Effectiveness	Effectiveness Themes	Barriers to Adoption	Barrier Themes
Zhuang et al.	pts value technology, improvement in memory, language, and visuospatial abilities	Improvement in cortical atrophy	Cost to acquire equipment, staff training, low reimbursement, time to administer tests	Cost
		Improved memory		Training
		Improved language		Low reimbursement
				Time of providers
Jelcic et al.	Improvement in memory, phonemic fluency, semantic fluency, stabilizing delayed/working memory	Improved MMSE scores (cognition)	Time of providers/staff on phone, training of staff, time to administer tests	Time of providers
		Improved language		Training
				Time of providers
Singh et al.	trials of isolated moderate-high intensity resistance training had significant effects on memory, cognition, and language	Improved resistance training	Cost to acquire equipment, staff training, low reimbursement, time to administer tests	Cost
		Improved MMSE scores (cognition)		Training
		Improved language		Low reimbursement
				Time of providers
Tarnanas et al.	improves untrained cognitive functions in MCI	Improved MMSE scores (cognition)	Cost to acquire equipment, staff training, low reimbursement, time to administer tests	Cost
				Training
				Low reimbursement
				Time of providers
Burdea et al.	Improvements in decision making and depression	Improved depression	Cost to acquire equipment, staff training, low reimbursement	Cost
				Training
				Low reimbursement
				Time of providers
Finn et al.	repetition-lag training (RLT), a form of recognition memory training reported	Improved memory	Cost to acquire equipment, staff training, low reimbursement, time to administer tests	Cost
				Training
				Low reimbursement
				Time of providers
Callan et al.	Improved task performance, in terms of speed, by nearly 50%	Improved MMSE scores (cognition)	Cost to acquire equipment, staff training, low reimbursement, time to administer tests	Cost
				Training
				Low reimbursement
				Time of providers
Cavallo et al.	Improvement in memory	Improved memory	Cost to acquire equipment, staff training, low reimbursement, time to administer tests	Cost
				Training
				Low reimbursement
				Time of providers
Hagovska et al.	improvement in postural reactions, attention, memory and language	Improved attention	Cost to acquire equipment, staff training, low reimbursement, time to administer tests	Cost
		Improved memory		Training
		Improved attention		Low reimbursement
		Improved language		Time of providers
Hyer et al.	improvement in mental sharpness	Improved MMSE scores (cognition)	Cost to acquire equipment, staff training, low reimbursement, time to administer tests	Cost
				Training
				Low reimbursement
				Time of providers
Boyd et al.	Improved brain waves	Improved EEG scores (brain waves)	dexterity limitations, use of touch screen and accidental screen presses, cost to acquire equipment, staff training, low reimbursement, time to administer benchmark tests	Dexterity limitations of older adults
				Cost
				Training
				Low reimbursement
				Time of providers
Yang et al.	Improvement in language, attention, calculation, verbal memory, and frontal function for the experimental group, convenience, savings in time	Improved MMSE scores (cognition)	Cost to acquire equipment, staff training, low reimbursement, time to administer tests	Cost
		Improved memory		Training
		Improved language		Low reimbursement
		Savings in time		Time of providers
Lee et al.	convenience, savings in time, improved cognition	Savings in time	Cost to acquire equipment, staff training, low reimbursement, time to administer tests	Cost
		Improved MMSE scores (cognition)		Training
				Low reimbursement
				Time of providers
Park et al.	Regarding health-related quality of life, the NCT group showed more improvement in vitality, role-emotional, and mental health compared with the CCT group	Improved attention	Cost to acquire equipment, staff training, low reimbursement, time to administer tests	Cost
		Improved memory		Training
		Improved vitality		Low reimbursement
		Improved mental health		Time of providers
		Improved quality of life		
Flak et al.	none	None reported	Cost to acquire equipment, staff training, low reimbursement, time to administer tests	Cost
				Training
				Low reimbursement
				Time of providers
Kahn	Increase in brain waves, increase in cognition, incresae in cerebral blood flow, improved neuro plasticity	Improved EEG scores (brain waves)	Cost to acquire equipment, staff training, low reimbursement, time to administer tests	Cost
		Improved MMSE scores (cognition)		Training
		Improved cerebral blood flow		Low reimbursement
		Improved neuro plasticity		Time of providers
Park	none	None reported	Cost to acquire equipment, staff training, low reimbursement	Cost
				Training
				Low reimbursement
				Time of providers
Park et al.	Improvement in physical outcomes, memory and brain stimulation	Improved vitality	Cost to acquire equipment, staff training, low reimbursement	Cost
		Improved memory		Training
		Improved EEG scores (brain waves)		Low reimbursement
				Time of providers
Robert et al.	significant differences in two attention tests	Improved attention	Cost to acquire equipment, staff training, low reimbursement	Cost
				Training
				Low reimbursement
				Time of providers
Thapa et al.	Intervention group exhibited a significantly improved executive function and brain function at the resting state	Improved EEG scores (brain waves)	Cost to acquire equipment, staff training, low reimbursement	Cost
				Training
				Low reimbursement
				Time of providers
Oliveria et al.	an improvement in overall cognitive function in the experimental group	Improved MMSE scores (cognition)	Cost to acquire equipment, staff training, low reimbursement	Cost
				Training
				Low reimbursement
				Time of providers
Seredakis et al.	No group interaction	None reported	Cost to acquire equipment, staff training, low reimbursement	Cost
				Training
				Low reimbursement
				Time of providers

## Appendix C

**Table A3:** Other observations incident to review.

Authors	Sample Size (#s only)	Bias within Study Selection Bias, Sample Bias, etc.	Effect Size	Country of Origin (Where the Study Was Conducted)	Statistics Used	Patient Satisfaction	Strength of Evidence	Quality of Evidence
Zhuang et al.	33	China only (selection bias), Mostly female (sample bias)	Not reported	China	Measures of central tendency, MANOVA, ANOVA, Wilk’s lambda	Positive effect on patient experience	I	A
		Short intervention period (design bias)				Pts value technology		
Jelcic et al.	27	Venice only (selection bias), Mostly female and Caucasian (sample bias)	not reported	Venice	Measures of central tendency, Kruskal–Wallis ANOVA, Mann–Whitney *U*-test	Positive effect on patient experience	II	B
Singh et al.	100	Australia and New Zealand only (selection bias)	small (0.2)	Australia and New Zealand	Measures of central tendency, Odds ratio	improved global cognitive function	I	A
Tarnanas et al.	114	Greece only (selection bias)	sensitivity 80.4%, specificity 94.3%Large effect (3.91)	Greece	Measures of central tendency, ANOVA	Positive effect on patient experience,	I	A
						pts value technology		
Burdea et al.	10	one country (selection bias), majority male (sample bias)	not reported	USA	paired *t*-test	high rates of satisfaction	II	B
Finn et al.	31	Sydney, Australia only (selection bias)	small (0.17)	Australia	Measures of central tendency, ANOVA, *t*-test	Positive effect on patient experience,	I	A
						pts value technology		
Callan et al.	27	Pittsburg, USA only (selection bias)	not reported	USA	Measures of central tendency, paired *t*-test, Fisher’s exact test	Positive effect on patient experience,	I	B
						pts value technology		
Cavallo et al.	80	Moncalieri, Italy (selection bias)	not reported	Italy	Measures of central tendency, repeated measures GLM, *t*-tests	Positive effect on patient experience,	I	A
						pts value technology		
Hagovska et al.	80	Kosice, Slovak Republic only (selection bias)	medium (0.64)	Slovakia	Measures of central tendency, ANOVA, *t*-tests, Shapiro–Wilk test, D’Agostino-Pearson test	Positive effect on patient experience,	I	A
						pts value technology		
Hyer et al.	68	US only (selection bias)	medium	USA	Measures of central tendency, ANOVA	pts value technology	II	A
Boyd et al.	19	Northern Ireland only (selection bias)	not reported	Ireland	Measures of central tendency, *t*-tests	Positive effect on patient experience,	III	B
						pts value technology		
Yang et al.	20	Namyangju, south Korea only (selection bias)	not reported	Korea	Measures of central tendency, Mann–Whitney *U*-test, *t*-tests	Positive effect on motivation and mood	I	B
						pts value technology		
Lee et al.	20	Chungbuk National University Hospital, Korea only (selection bias)	not reported	Korea	Measures of central tendency, independent *t*-test, Mann–Whitney *U*-test	not reported	I	B
		limited number of treatment sessions (design bias)				pts value technology		
Park et al.	78	one country (selection bias)	not reported	Korea	Measures of central tendency	not reported	I	A
						pts value technology		
Flak et al.	68	Norway only (selection bias), majority male (sample bias)	Not reported	Norway	Linear mixed models	patients experienced frustration	I	A
Kahn	38	Pakistan only (selection bias)	not reported	Pakistan	ANOVA with Scheffe post hoc analysis, paired *t*-test	not reported	I	A
Park	21	Korea only (selection bias)	not reported	Korea	ANOVA with Shapiro–Wilks test, student’s *t*-test	not reported	I	A
Park et al.	45	One country (selection bias)	not reported	Korea	GLM	not reported	III	A
Robert et al.	46	One country (selection bias)	not reported	France	Student *t*-test, Wilcoxon-Mann–Whitney, Chi-square, Fisher’s exact, and Wilcoxon	not reported	I	A
Thapa et al.	66	One country (selection bias)	not reported	Korea	ANOVA, Shapiro–Wilk	not reported	I	A
Oliveria et al.	34	One country (selection bias)	large	Portugal	ANOVA with Bonferroni correction	not reported	I	A
Seredakis et al.	43	One country (selection bias)	medium	Australia	Chi-square, Shapiro–Wilk, Wilcoxon signed rank test, Mann–Whitney U test	not reported	II	A
